# Aspects epidemiologiques, cliniques et therapeutiques des otites externes: à propos de 801 cas

**DOI:** 10.11604/pamj.2014.17.142.3735

**Published:** 2014-02-28

**Authors:** Amana Bathokedeou, Pegbessou Essobozou, Patassi Akouda, Boko Essohanam, Kpémissi Eyawelohn

**Affiliations:** 1 Service ORL et CCF du CHU Tokoin, Togo; 2 Service de Maladies infectieuses du CHU Tokoin, Togo; 3 Service ORL et CCF du CHU Campus, Togo

**Keywords:** Otite externe, épidémiologie, complications, microbiologie, otitis externa, epidemiology, complications, microbiology

## Abstract

L'objectif de ce travail etait de déterminer l’épidémiologie, la clinique et la thérapeutique des otites externes (OE). Il s'agissait d'une **é**tude rétrospective d'une année menée du 1^er^ janvier au 31 décembre 2006 dans le service ORL du CHU-Tokoin. Huit cent un cas d'otite externe (OE) soit 11,9% des consultations étaient recensés. Le sexe féminin représentait 476 cas (59,42%). Le sex ratio était de 0,68. L’âge moyen des patients était de 25,4 ans avec des extrêmes de 05 mois et 81 ans. La tranche d’âge de 0-15 ans était la plus fréquente avec 360 cas (45%). L'allergie dans 74 cas (60,66%), la lésion de grattage dans 24 cas (19,67%), les corps étrangers du conduit auditif externe dans 18 cas (14,75%) et la natation dans 6 cas (4,92%) étaient les facteurs favorisants. L'otalgie dans 638 cas (79,65%) était le symptôme le plus fréquent. Les différentes formes cliniques des otites externes se répartissaient comme suit: otite externe diffuse dans 612 cas (76,40%), furoncle du CAE 126 dans cas (15,73%), otomycose dans 58 cas (7,24%), zona du conduit auditif externe dans 3 cas (0,37%) et otite externe nécrosante dans 2 cas (0,25%). Les gouttes auriculaires étaient administrées à tous les patients. L’évolution avait noté 799 patients (99,75%) guéris sans complication, un cas de décès et un cas de guérison avec séquelle. Traitée correctement, l'otite externe guérit sans complication. Son éviction passe par une sensibilisation des populations sur l'entretien du conduit auditif externe.

## Introduction

Les otites externes (OE) sont des inflammations ou des infections de l'oreille externe [1]. Elles découlent d'une altération de l’équilibre du conduit auditif externe(CAE). L'otite externe est l'otite la plus fréquente. Elle touche les enfants et les adultes. Elle est souvent secondaire à des microtraumatismes, à une macération, à un eczéma ou à un corps étranger du conduit auditif externe. Plusieurs formes cliniques peuvent se présenter selon l'agent infectieux et selon le terrain. On distingue: l'otite externe diffuse, le furoncle du CAE, l'otomycose, l'otite externe phlycténulaire et l'otite externe nécrosante. Le diagnostic est clinique et le traitement simple sauf pour l'otite externe nécrosante qui est la forme redoutable des OE. Pour améliorer la prise en charge de cette pathologie dans le service, Cette étude a été entreprise dans le but de déterminer l’épidémiologie, la clinique et la thérapeutique des OE.

## Méthodes

Il s'agissait d'une étude rétrospective d'un an menée du 1^er^ Janvier au 31 Décembre 2006 dans le service ORL du CHU-Sylvanuis Olympio. Elle a portée sur des dossiers des patients ayant présentés une otite externe au cours de la période d’étude sans distinction d’âge, ni de sexe. L'otite externe est diagnostiquée chez tout patient dont les signes fonctionnels se situent aux oreilles, avec un examen clinique retrouvant une douleur à la traction du pavillon ou à la pression du tragus et une altération de la peau du conduit auditif externe. N’étaient pas inclus dans cette étude les patients dont les dossiers étaient incomplets. Les paramètres épidémiologiques, cliniques, thérapeutiques et évolutifs étaient étudiés.

## Résultats

Sur le plan épidémiologique, 6732 patients avaient consulté au cours de la période d’étude; dont 801cas d'OE soit 11,9%. Le sexe féminin représentait 476 cas (59,42%) contre 325 cas pour le sexe masculin (40,58%). Le sex ratio était de 0,68. L’âge moyen des patients était 25,4 ans avec des extrêmes de 05 mois et 81 ans. La tranche d’âge de 0-15 ans était la plus fréquente avec 360 cas (45%) (Tableau 1). Cent vingt-deux patients (15,23%) avaient signalés un facteur favorisant. L'allergie dans 74 cas (60,66%), la lésion de grattage dans 24 cas (19,67%), les corps étrangers du conduit auditif dans 18 cas (14,75%) et la natation dans 6 cas (4,92%) étaient les facteurs favorisants.

**Tableau 1:** Répartition des patients selon l’âge

	Effectif	pourcentage(%)
0-15 ans	360	44,95
16-30 ans	157	19,60
31-45 ans	138	17,23
46-60 ans	112	13,98
>60 ans	34	4,24
**Total**	801	100

Plusieurs motifs de consultations avaient été notés: otalgie dans 638 cas (79,65%) était le symptôme le plus fréquent (Tableau 2). L'otite externe était unilatérale chez tous patients et l'oreille droite était la plus concernée dans 542 cas (67,66%). Les différentes formes cliniques des otites externes se répartissaient comme suit: otite externe diffuse dans 612 cas (76,40%), furoncle du CAE 126 dans cas (15,73%), otomycose dans 58 cas dans (7,24%), zona du conduit auditif externe dans 3 cas (0,37%) et 2 cas (0,25%) d'otite externe nécrosante survenue chez deux patients séropositifs dont un était sous antirétroviraux (ARV).

**Tableau 2:** Répartition des différents motifs de consultation

	Effectif	Pourcentage(%)
Otalgie	638	79,65
Otorrhée	125	15,60
Prurit	10	1,25
Otorrhée + otalgie	6	0,75
Otalgie + hypoacousie	15	1,90
Otalgie + otorrhée + prurit	4	0,45
Acouphène + prurit + otalgie	3	0,40
**Total**	801	100

Les gouttes auriculaires contenant une association d'antibiotique et corticoïde étaient administrées à tous les patients une otite externe diffuse ou un furoncle du CAE. Lorsque le tympan n’était pas visualisé, les gouttes auriculaires utilisées étaient non ototoxiques (sans aminosides, ni de corticoïdes) en association avec des antalgiques et des anti-inflammatoires par voie orale. Pour les otomycoses, les gouttes auriculaires contenaient des antifongiques. Dans le cas du zona auriculaire le traitement était symptomatique. Les deux cas d'otite externe nécrosante étaient hospitalisés et avaient bénéficié d'un traitement chirurgical et une antibiothérapie par voie parentérale.

**Figure 1:**
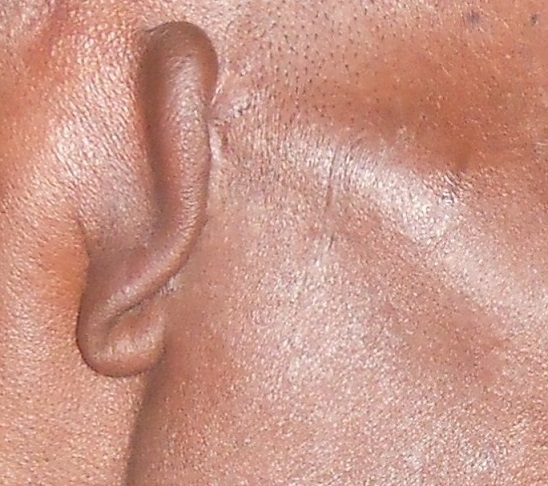
Séquelle de l'otite externe nécrosante

**Figure 2:**
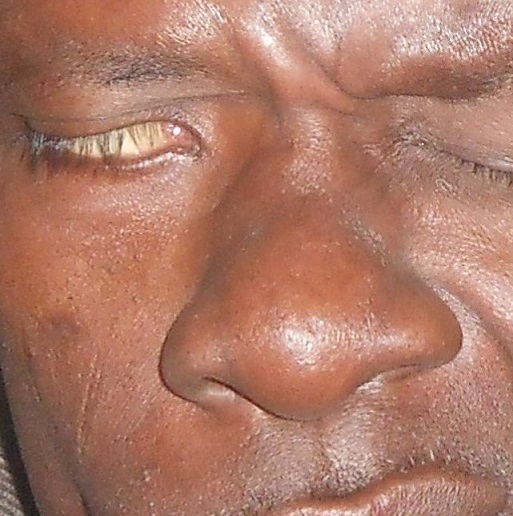
Paralysie faciale périphérique droite

L’évolution était marquée par le décès du patient qui présentait une otite externe nécrosante qui n’était pas sous ARV au deuxième jour d'hospitalisation, un cas de séquelle à type de paralysie faciale périphérique associée à une sténose du CAE noté chez la deuxième otite externe nécrosante (Figure 1, Figure 2). Tous les autres patients avaient guéri sous ces traitements. Huit patients (1%) avaient récidivé au cours de la même année.

## Discussion

La prévalence des OE varie selon les études, elle est de 11,9% dans cette étude, ce résultat est proche de celui retrouvés aux USA [2]. L'OE atteint tous les individus à n'importe quel âge, cet âge se situait entre 05 mois et 81 ans dans cette étude. La survenue d'une OE chez un sujet est le résultat d'un mauvais entretien du CAE; qui à l’état normal est protégé par le cérumen [2], ce qui restaure un équilibre entre le conduit et les germes saprophytiques. Les facteurs favorisants dénaturant cet équilibre de cette étude sont identiques aux données de la littérature [2, 3]. L'humidité du CAE est l'un de ces facteurs favorisant des OE [2], elle se retrouve la plupart du temps chez des nageurs, ou chez d'autres sujets en période de baignade en été. Ce fait est à l'origine du nom « swimmer's ear » que les auteurs anglais donnent à l'OE [2]. Ces faits n'ont pas été formellement identifiés dans cette étude. L'allergie naso-sinusienne par le prurit auriculaire qu'elle occasionne est à l'origine des microtraumatismes du CAE responsables des OE.

L'otalgie était le motif de consultation le plus fréquent de cette étude, ceci est conforme aux données de la littérature [2]. D'autres signes prurit, hypoacousie, acouphène, l'otorrhée font partie des motifs de consultation. La douleur à la traction du pavillon ou à la pression du tragus permet de poser le diagnostic d'une OE. Les examens paracliniques ne sont pas nécessaires au diagnostic sauf en cas de complications ou si on envisage de faire une étude bactériologique. Les agents infectieux sont bactériens, viraux, fongiques. La fréquence des bactéries dans les OE est variable suivant des études et des régions; P. aeruginosa est retrouvé aux USA et Royaume uni [1, 2, 4, 5], *S. aureus* en Taiwan et en Norvège [6, 7]. Ces deux bactéries sont saprophytes de la flore du CAE [2].

L'usage des gouttes auriculaires est de règle dans le traitement des OE [2]. Suivant la sensibilité des germes ces gouttes contiennent soit une associations antibiotiques (aminosides, fluoroquinolones, polypeptides) et corticoïdes, ou antibiotiques seuls [2]. L'utilisation abusive des gouttes auriculaires à base d'antibiotique et de corticoïdes pourrait être à l'origine de l'otomycoses [3].

L'OE guérit sous traitement, avec quelques épisodes de récidives pouvant entrainer une évolution vers la chronicité [2]. Dans notre étude seulement 1% des patients avait une récidive. L'otite externe nécrosante est la complication redoutable de l'OE. Elle survient chez des sujets en immunodépression [2]. Elle est souvent causée par P.aeruginosa [8], mais les champignons peuvent également en être la cause [9, 10]. Les autres complications peuvent être une cellulite, une parotidite, une périchondrite voire une sténose du CAE [2].

## Conclusion

L'otite externe est une pathologie relativement fréquente dans notre pratique. Traitée correctement, elle guérit sans complications sauf pour l'otite externe nécrosante qui nécessite un diagnostic précoce et prise en charge rapide. Son éviction passe par une sensibilisation des populations sur l'entretien du CAE.
